# Pathogenic variants in *GBX2* cause craniofacial microsomia

**DOI:** 10.1016/j.gendis.2025.101814

**Published:** 2025-08-21

**Authors:** Run Yang, Wenqing Han, Liang Wang, Xin Chen, Ying Chen, Bowen Li, Maoxiang Qian, Dong Liu, Tianyu Zhang, Jing Ma

**Affiliations:** a ENT Institute, Department of Facial Plastic and Reconstructive Surgery, Eye & ENT Hospital, Fudan University, Shanghai 200031, China; b Institute of Special Environmental Medicine, Nantong University, Nantong, Jiangsu 226001, China; c Department of Stomatology, Affiliated Hospital of Nantong University, Nantong, Jiangsu 226001, China; d Institute of Pediatrics and Department of Hematology and Oncology, Children’s Hospital of Fudan University, National Children’s Medical Center, and the Shanghai Key Laboratory of Medical Epigenetics, International Co-Laboratory of Medical Epigenetics and Metabolism (Ministry of Science and Technology), Institutes of Biomedical Sciences, Fudan University, Shanghai 200032, China; e School of Life Science, Nantong Laboratory of Development and Diseases, and Co-Innovation Center of Neuroregeneration, Nantong University, Nantong, Jiangsu 226001, China; f NHC Key Laboratory of Hearing Medicine, Fudan University, Shanghai 200031, China; g Institute of Medical Genetics & Genomics, Fudan University, Shanghai 200032, China

Craniofacial microsomia (CFM) is a congenital malformation resulting from abnormal development of cranial neural crest cells (CNCCs) and the first and second pharyngeal arches, affecting an estimated 1 in 3600 to 5600 live births. CFM encompasses a spectrum of abnormalities, ranging from isolated microtia to maxillary and mandibular hypoplasia, facial asymmetry, underdevelopment of the orbit, facial soft tissue, and/or facial nerve. The role of genetic factors in the occurrence of CFM is well-established. Although most cases of CFM are sporadic, both autosomal dominant and recessive inheritance patterns have been observed. Several genes, including *SF3B2*, *HOXA2* and *FOXI3*, have been identified as pathogenic contributors to CFM.^1^ Haploinsufficiency of *SF3B2* accounts for approximately 3% of sporadic CFM cases and about 25% of familial cases. To date, six families with microtia caused by *HOXA2* variants have been reported. Additionally, approximately 3.1% of CFM cases are associated with pathogenic variants of *FOXI3*. Despite the identification of several causative genes, the genetic etiology of CFM remains unresolved for a significant proportion of cases.

In 2016, a genome-wide association study identified 13 susceptibility loci for CFM, one of which harbors the gastrulation brain homeobox 2 (*GBX2*) gene.^2^ GBX2 is essential for the migration and survival of a subpopulation of CNCCs. In *Gbx2* deficient mice, which die shortly after birth, severe malformations of the middle ear, including CNCCs-derived structures such as the malleus, stapes, and incus, are observed. Despite these findings, no specific variants in *GBX2* have been reported in CFM, and the role of *GBX2*as a pathogenic gene for CFM had not been established.

Through whole-exome sequencing (WES) of a cohort comprising 201 families clinically diagnosed with CFM, we identified two rare heterozygous missense variants in *GBX2*: c.11C > T (p. Ala4Val) and c.201G > C (p. Gln67His), segregating across three unrelated CFM families. The c.11C > T p. Ala4Val variant was found in pedigree 1 and 2, while c.201G > C p. Gln67His variant was identified in pedigree 3. Manifestations of patients with CFM carried the *GBX2* variant in pedigree 1, 2 and 3 were presented in the footnote of Table S1. The *GBX2* variants co-segregated with the CFM phenotype in all three pedigrees, following an autosomal dominant inheritance pattern (Fig. 1A–C; Fig. S1 and Table S1). Conservation analysis indicated that the amino acids at both variant sites are highly conserved among vertebrates (Fig. S2A). Both variants are located in exon 1 of *GBX2*, and neither resides within known functional domains of the GBX2 protein (Fig. S2A; Fig. 1D). Pathogenicity predictions suggest that both variants are disease-causing (Table S2). Structural modeling suggested that the p. Ala4Val substitution may perturb protein folding due to steric hindrance caused by the bulkier valine residue, whereas p. Gln67His, although affecting a less conserved region, also compromises protein activity^3^ (Fig. S2B).

**Figure 1:**
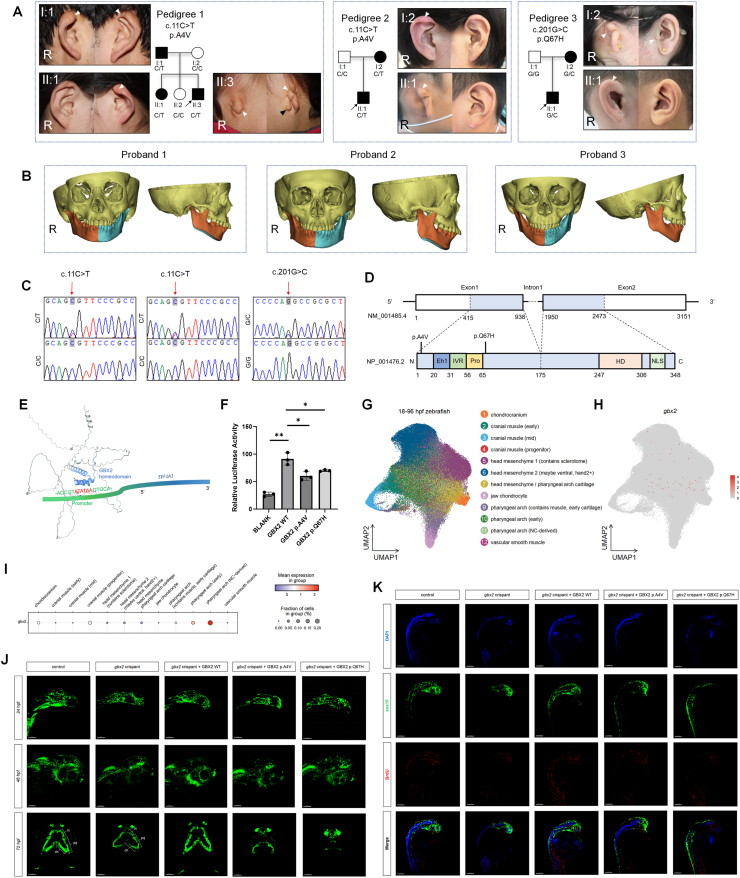
Pathogenic variants in GBX2 cause craniofacial microsomia. **(A)** Pedigrees with CFM: Squares represent male individuals, while circles represent female individuals. Shaded symbols indicate affected individuals, with a black arrow marking the proband. White arrows specifically denote auricle malformations. R, right. **(B)** 3D-CT reconstruction revealing mild mandibular asymmetry in probands 1 and 2, with blue and orange indicating the left and right hemi-mandibles. In contrast, proband 3 does not exhibit mandibular asymmetry. R, right. **(C)** Sanger sequencing electropherograms of the three pedigrees with CFM with red arrow indicating the mutation site. **(D)** The exon-intron structure of the *GBX2* gene (upper) and the known functional domains of the GBX2 protein (below). The positions of the two mutations are marked on the protein structure diagram. Solid box: coding exons, open box: non-coding exons. **(E)** Schematic representation of the GBX2 homeodomain binding to the ATATAA motif in the *EEF1A1* core promotor. **(F)** Effects of the two mutations on the transactivation of EEF1A1 of wild-type and mutant GBX2 in HEK293T cells. **(G)** UMAP plot of zebrafish single-cell transcriptome data depicting all cells types from the pharyngeal arch lineage from 18 hpf to 96 hpf. **(H)** Feature plot illustrating the expression distribution for *gbx2* in cells from the pharyngeal arch lineage, where expression levels are color-coded and overlaid onto the UMAP plot. **(I)** Dot plot depicting *gbx2* in cells from the pharyngeal arch lineage. **(J)** Maximum projections confocal live image of *Tg(sox10:* GFP) zebrafish at 24, 48 and 72hpf. Scale bar = 300 μm. **(K)** Lateral views of *Tg(sox10:* GFP) zebrafish at 24 hpf, with nuclei labeled with DAPI and proliferating cells labeled with BrdU. Scale bar = 300 μm.

Given that GBX2 functions as a transcription factor, we further evaluated the impact of these variants on its ability to activate transcription of downstream target genes. Based on Chromatin immunoprecipitation sequencing (ChIP-seq) data,^4^ the homeodomain of GBX2 binds to the core promoter of the eukaryotic translation elongation factor 1 alpha 1 (*EEF1A1*) gene to promote its expression in HEK-293T cells (Fig. 1E). We co-transfected HEK-293T cells with wild-type or mutant *GBX2* with an *EEF1A1* core promoter luciferase reporter (Fig. S2C–E). Luciferase assays revealed that both p. Ala4Val and p. Gln67His significantly reduced *EEF1A1* promoter activity compared to wild-type, indicating impaired transcriptional activation (Fig. 1F). These findings support a loss-of-function mechanism.

To assess the developmental significance of GBX2 *in vivo*, we employed a zebrafish model, leveraging its short development cycle and embryo transparency. The zebrafish pharyngeal arch cartilage, including Meckel’s cartilages, the palatoquadrate, and ceratohyal, is evolutionarily homologous to human external and middle ear.^5^ Zebrafish CNCCs arise from the neuroepithelium and migrate to the pharyngeal arches, where they differentiate into craniofacial cartilages. The zebrafish *gbx2* shares 60.1% nucleotide and 72.1% amino acid identity with the human ortholog, and phylogenetic analysis confirms its conservation across vertebrates (Fig. S3A–C).

Prior to functional validation, we analyzed *gbx2* expression patterns during zebrafish embryogenesis using the zebrafish embryogenesis spatiotemporal transcriptomic atlas (ZESTA). *gbx2* was detected as early as 3.3 hpf in marginal blastomeres and later localized to the neural keel and cranial neural crest by 18 hpf (Fig. S3D). Single-cell analysis from 18 to 96 hpf confirmed that *gbx2* expression was largely restricted to the neural crest and early pharyngeal arch lineages (Fig. 1G–I; Fig. S3E). These findings support the hypothesis that *GBX2* functions in CNCC-driven craniofacial development.

Using CRISPR interference (CRISPRi), *gbx2* was knocked down in *Tg(sox10:GFP)* zebrafish embryos (crispants), and qPCR confirmed efficient suppression of gene expression (Fig. S4A). The overall development of the *gbx2* knockdown zebrafish appeared normal, as assessed by body length and the ratio of head thickness to body length (Fig. S4B–D).

While CNCC numbers were comparable between control and knockdown groups at 24 hpf, a significant reduction was evident by 48 hpf (Fig. 1J). By 72 hpf, these embryos exhibited hypoplasia of pharyngeal arch cartilages (Fig. 1J). These observations indicate that *GBX2* is essential for CNCC proliferation and the subsequent development of craniofacial skeletal elements.

Rescue experiments were performed by co-injecting wild-type or mutant human *GBX2* mRNA into *gbx2*-deficient zebrafish embryos. Wild-type *GBX2* successfully restored both CNCC numbers and cartilage morphology, whereas neither p.Ala4Val nor p.Gln67His was able to rescue the phenotypes (Fig. 1J). Notably, the p.Ala4Val variant exhibited a more pronounced failure to rescue, which parallels the more severe clinical features observed in carriers of this variant. Quantitative imaging and fluorescence intensity analysis of *sox10-GFP* further substantiated these differences (Fig. S4E–G), emphasizing the functional divergence between the two alleles.

Moreover, BrdU assays at 24 hpf demonstrated a marked reduction in proliferating CNCCs in *gbx2*-deficient embryos, which could be rescued by wild-type but not mutant *GBX2* mRNA (Fig. 1K; Fig. S4H). These results underscore the essential role of *GBX2* in supporting CNCC proliferation and suggest that the observed phenotypic defects originate from early proliferative deficiencies. These findings establish a direct mechanistic link between *GBX2* function and craniofacial morphogenesis.

These results illustrate that *gbx2* knockdown inhibits CNCC proliferation, leading to a reduction of CNCC numbers and insufficient materials for pharyngeal arch cartilage formation in zebrafish. This implicates its disruption in pathogenesis in CFM. Additionally, the findings further demonstrate that the two novel variants of *GBX2* are loss-of-function variants.

In this study, we analyzed WES data from 201 families affected by CFM and identified two missense variants in the *GBX2* gene across three unrelated families. By integrating *in vitro* pathogenicity assessments and *in vivo* phenotypic analysis, our research highlighted the pathogenic nature of the variants and underscored the critical role of GBX2 in the proliferation of CNCCs. This finding provides functional validation supporting the pathogenic role of *GBX2* in CFM, which was previously implicated through GWAS. Our data suggest that approximately 1.5% of CFM cases can be attributed to variants in *GBX2*. Further studies with larger cohorts are necessary to better determine the overall contribution of *GBX2* variants to CFM. Secondly, although our zebrafish model recapitulated craniofacial features, discrepancies with human phenotypes remain, indicating the need for better models. Third, while GBX2 affects CNCC proliferation, its role in migration remains unclear. Finally, phenotypic variability among individuals with the same variant suggests additional genetic or epigenetic modifiers yet to be identified.

## CRediT authorship contribution statement

**Run Yang:** Writing – review & editing, Writing – original draft, Methodology, Investigation, Formal analysis, Data curation, Conceptualization. **Wenqing Han:** Writing – review & editing, Writing – original draft, Methodology, Investigation, Formal analysis, Data curation, Conceptualization. **Liang Wang:** Methodology, Investigation, Formal analysis, Data curation. **Xin Chen:** Resources, Investigation. **Ying Chen:** Resources, Investigation. **Bowen Li:** Visualization. **Maoxiang Qian:** Data curation. **Dong Liu:** Resources, Project administration, Conceptualization. **Tianyu Zhang:** Supervision, Project administration, Funding acquisition. **Jing Ma:** Writing – review & editing, Supervision, Project administration, Funding acquisition.

## Ethics declaration

All procedures involving human participants complied with the World Medical Association’s Declaration of Helsinki and received approval from the Institutional Research Ethics Committee of the Eye & ENT Hospital, Fudan University (Approval No. 2020069). Written informed consent for clinical and biological investigations was obtained from all participants or their legal guardians.

## Data availability

All data are available upon request.

## Funding

This work was supported by the 10.13039/501100001809National Natural Science Foundation of China (Nos. 82371173, 82271889), and the Science and Technology Innovation Plan of Shanghai Science and Technology Commission (China) (No. 23ZR1409400).

## Conflict of interests

No conflict of interests has been declared by the authors.
