# Supplementary material for: Pathogenic variants in GBX2 cause craniofacial microsomia
Source: Genes Dis. 2025 Aug 21;13(4):101814. doi: 10.1016/j.gendis.2025.101814 (PMC12999328; doi:10.1016/j.gendis.2025.101814)
Supplement: Multimedia component 1 [file mmc1.docx]

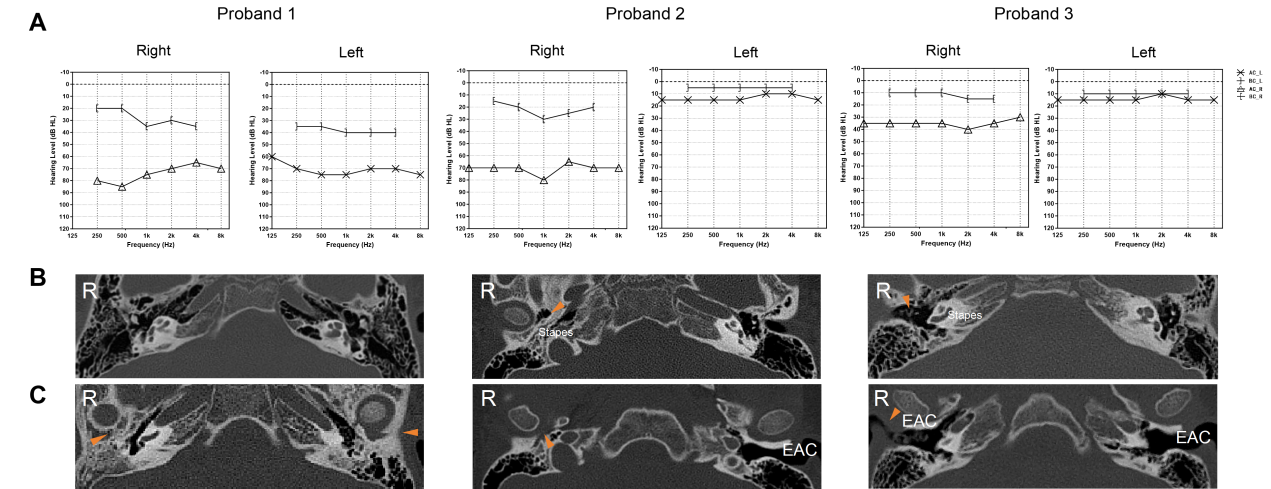


**Figure S1** Clinical examination of probands from craniofacial microsomia families. **(A)** The audiograms showed varying degrees of hearing loss in all three probands. **(B, C)** CT images displayed different extents of external auditory canal abnormalities in all three probands, with middle ear involvement noted in probands 2 and 3. The red arrows specifically denote involvement of the ossicular chain. R, right.


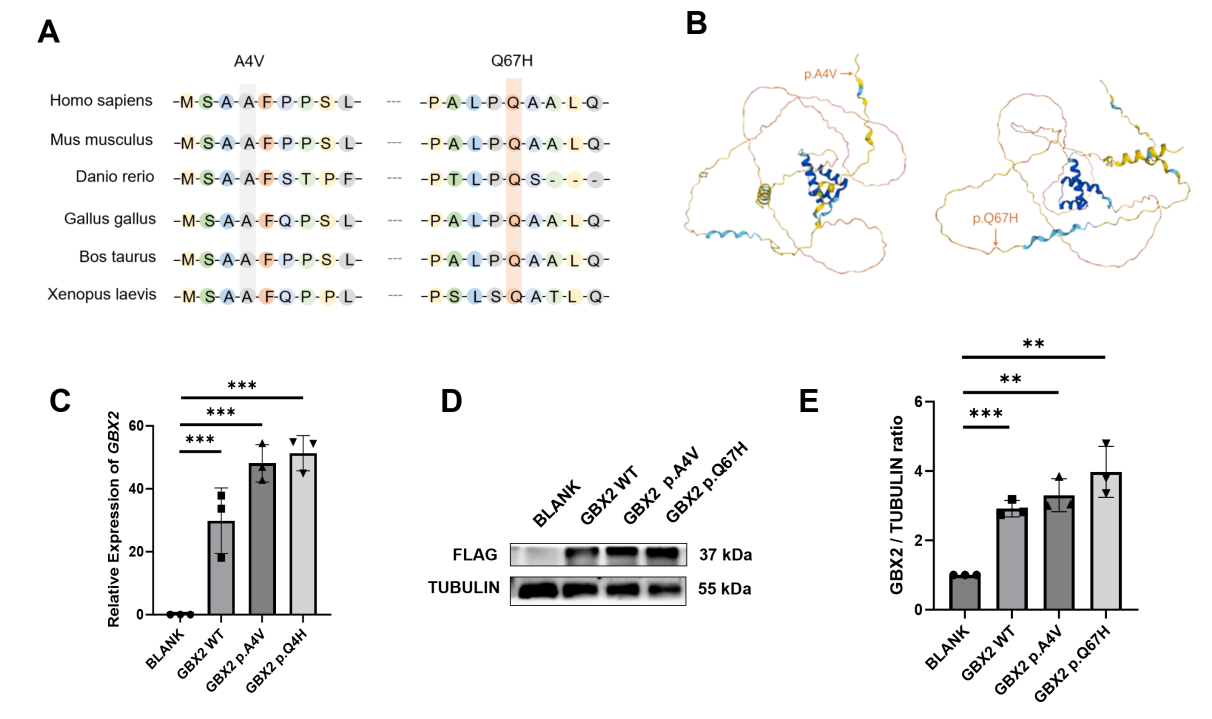


**Figure S2** The pathogenicity analysis of the two *GBX2* mutations. **(A)** Alignment of GBX2 amino acids. The two mutated amino acids are highly conserved among different species (highlighted in boxes). **(B)** AlphaFold-predicted structures of *GBX2* variants: p. Ala4Val (left) and p. Gln67His (right), showing localized structural alterations at the respective mutation sites. **(C)** GBX2 overexpression in HEK-293T cells was validated by quantitative PCR. **(D)** Expression of GBX2 WT and mutant flag-GBX2 vectors in HEK-293T cells was validated by western blotting. **(E)** Quantitative analysis of western blotting in (D).


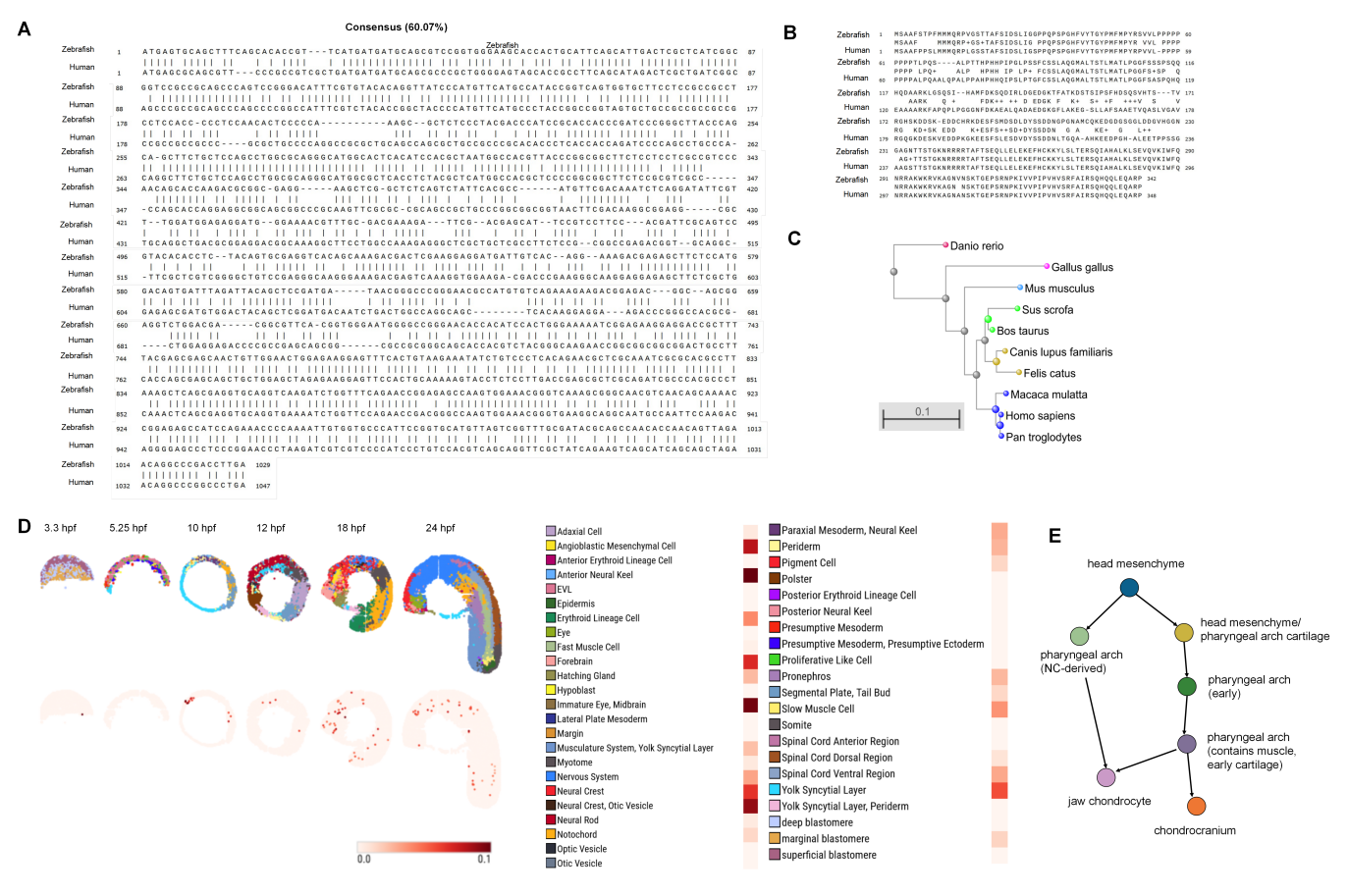


**Figure S3** Expression analysis of *gbx2* in zebrafish. **(A)** Nucleotide coding sequence alignment between zebrafish *gbx2* (NM_152964.1) and human *GBX2* (NM_001485.4). **(B)** Amino acid sequence alignment of zebrafish Gbx2 with human GBX2 protein. **(C)** A phylogenetic tree was constructed using protein sequences from different species, with a scale bar indicating 0.1 amino acid replacements per site. **(D)** Spatiotemporal expression patterns of *gbx2* across of zebrafish embryo across six developmental stages, with a red gradient indicating expression intensity from 0.0 to 0.1. **(E)** Graphical representation of cell types from the pharyngeal arch lineage from 18 hpf to 96 hpf, based on lineage relationships documented in ZFIN.


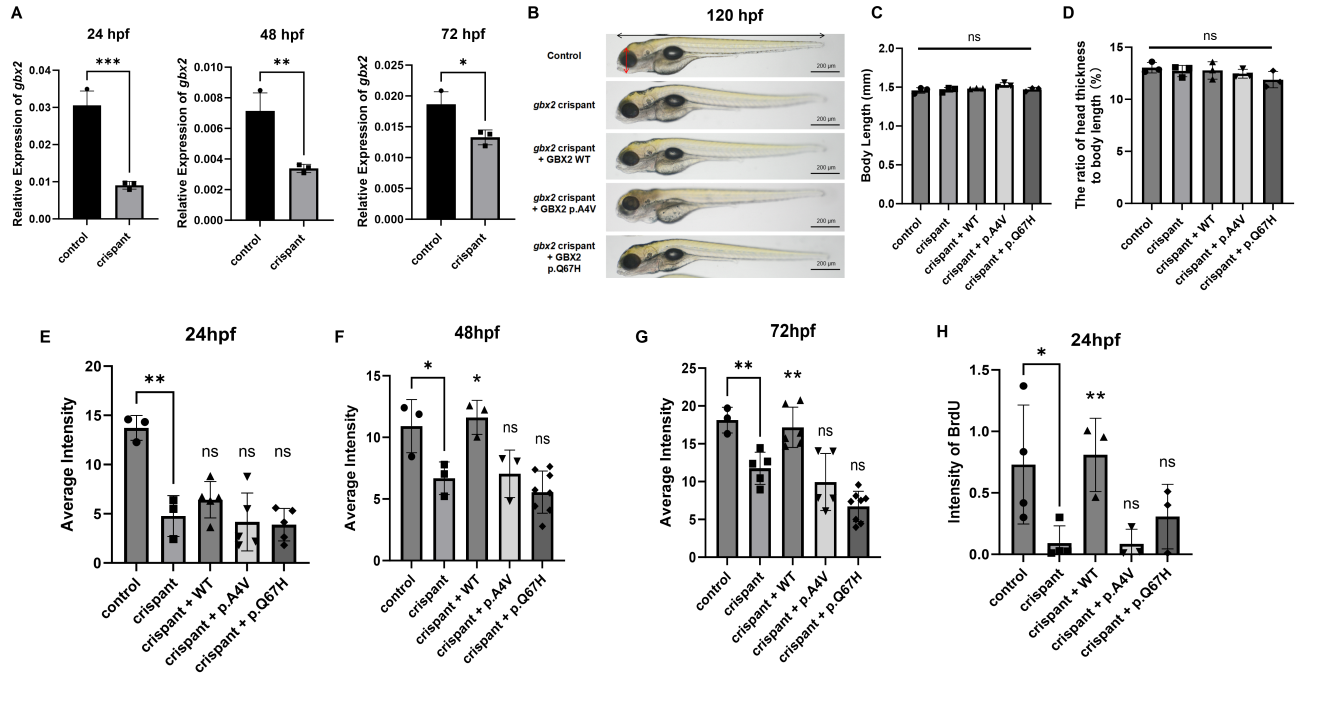


**Figure S4** Effects of *gbx2* deficiency in zebrafish. **(A)** The quantitative PCR assay demonstrated a significant reduction in *gbx2* expression in zebrafish crispants compared with the control group (*n* = 3, two-tailed *t*-test). **(B)** Overall development of the zebrafish was observed under a dissecting microscope. A schematic diagram depicting body length (black arrow) and head thickness (red arrow) was used to calculate the ratio of head thickness to body length. Scale bar, 200 μm. **(C, D)** Quantitative analysis of body length (C) and relative head thickness to the body length (D) of zebrafish at 120 hpf (*n* = 3, two-tailed *t*-test). **(E–H)** Quantitative analysis of relative sox10 (E–G) and BrdU (H) fluorescence intensity in *Tg(sox10:*GFP*)* zebrafish (*n* ≥ 3, two-tailed *t*-test).
