# Supplementary material for: Pathogenic variants in GBX2 cause craniofacial microsomia
Source: Genes Dis. 2025 Aug 21;13(4):101814. doi: 10.1016/j.gendis.2025.101814 (PMC12999328; doi:10.1016/j.gendis.2025.101814)
Supplement: Multimedia component 2 [file mmc2.docx]

**SUPPLEMENTARY METHODS AND MATERIALS**

**Patient cohort**

Between June 2020 and June 2021, a total of 201 pedigrees with a proband diagnosed with craniofacial microsomia (CFM) were evaluated at the Department of Facial Plastic and Reconstructive Surgery, Eye & ENT Hospital, Fudan University. All procedures involving human participants complied with the World Medical Association's Declaration of Helsinki and received approval from the Institutional Research Ethics Committee of the Eye & ENT Hospital, Fudan University (Approval No. 2020069). Written informed consent for clinical and biological investigations was obtained from all participants or their legal guardians.

**Ethics Declaration**

All procedures involving human participants complied with the World Medical Association's Declaration of Helsinki and received approval from the Institutional Research Ethics Committee of the Eye & ENT Hospital, Fudan University (Approval No. 2020069). Written informed consent for clinical and biological investigations was obtained from all participants or their legal guardians.

**Demographic and clinical examinations**

Demographic data, clinical photographs and medical histories were collected from all pedigrees. The classification of CFM was based on the orbit, mandible, ear, nerve, and soft tissue (OMENS) system (Table S2), with each component rated on a scale of 0 to 3^1^. Microtia was classified into four grades (I–IV) according to Marx's criteria^2^ . Each proband underwent comprehensive clinical evaluations, which included detailed physical examinations, otoscopic assessment, and pure-tone audiometry. Additionally, craniofacial three-dimensional computed tomography (3D-CT) scans were performed using a multi-detector row helical CT scanner (Philips, Eindhoven, Netherlands) with the following parameters: 120 kVp, 400 mAs, a scan time of 750 ms, and a matrix size of 512×512. The Digital Imaging and Communications in Medicine datasets were imported into Mimics software (Materialise, Leuven, Belgium) for further analysis. Grayscale threshold values in Hounsfield units were set to accurately delineate the boundaries and properties of the mandible. A 3D digital model of the mandible was generated and exported in standard tessellation language file format. To assess mandibular asymmetry, the 3D model was segmented along the sagittal plane, and the asymmetry index (AI) was calculated using the following formula, where V denotes the volume of the mandible.


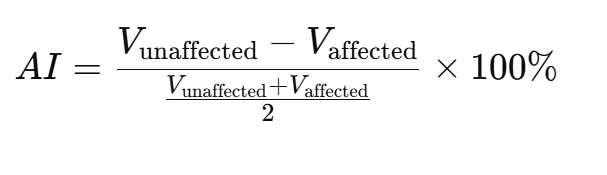


**Whole Exome Sequencing**

Peripheral blood samples were collected from individuals using BD Vacutainer® EDTA anticoagulant tubes (Becton, NJ, USA) and stored at -20°C. Genomic DNA was extracted from these samples using the DNeasy 96 Blood & Tissue Kit (Qiagen, Hombrechtikon, Switzerland). The quality of the extracted genomic DNA was assessed with the CapTruth Human Exome V2.0 kit (Nantong ZhongKe Medical Laboratory, Jiangsu, China). DNA was subsequently fragmented, purified with the Ampure XP system (Beckman Coulter, Beverly, USA), and quantified with Qubit BR ssDNA kit (Agilent Technologies, Waldbronn, Germany). Paired-end sequencing with150 bp reads was performed on the DNBSEQ-T7 platform (Nantong ZhongKe Medical Laboratory, Jiangsu, China). Sequencing reads were processed using Illumina Base-calling software and stored in FASTQ format for downstream analysis. The sequencing data for each sample were aligned to the human reference genome (GRCh37/hg19) using the Burrows-Wheeler Aligner^3^. Variant calling was conducted with the Genome Analysis Toolkit^4^ and CNVkit^5^ . Variants were annotated using ANNOVAR^6^ and SnpEff^7^. Variants that were synonymous, located in segment duplications, or had a minor allele frequency greater than 0.01 were excluded from further analysis. Pathogenicity prediction was made using Mutation Taster 2021^8^, Polyphen2^9^, and CADD^10^. These predictions were based on data from the OMIM (https://omim.org) and ClinVar (https://www.ncbi.nlm.nih.gov/clinvar) databases to select candidate genes. We identified three families in which seven individuals carried rare and potentially disease-causing variants in the *GBX2* gene, using the reference sequence NM_001485.4.

**Sanger Sequencing**

Validation of variant sites was performed on peripheral blood samples using Sanger sequencing. Specific primers targeting the variant sites were designed using Primer Primer 5.0 software (Table S3). The 50 µL polymerase chain reaction (PCR) reaction system included: 3 µL genomic DNA, 2 µL of each 10 µmol/L forward and reverse primer, 25 µL of PCR premix (Vazyme Biotech Co., Ltd., Nanjing), and 18 µL of ddH_2_O. The amplification protocol was as follow: initial denaturation at 95°C for 3 minutes, followed by 35 cycles of 95°C for 30 seconds (denaturation), 58°C for 20 seconds (annealing), and 72°C for 30 seconds (extension), with a final extension at 72°C for 3 minutes. The PCR products were purified and sequenced by Sanger sequencing (Nantong Zhongke Medical Laboratory, Jiangsu, China). Sequence alignment and analysis were conducted using SeqMan software (https://www.dnastar.com/software/seqman-pro), and electropherograms were visualized with Chromas 2.6.6 (http://technelysium.com.au/wp/chromas).

To assess the conservation of the mutated sites, sequences were downloaded from the NCBI Gene database (https://www.ncbi.nlm.nih.gov/gene). Multiple sequence alignments of *GBX2* across various species were performed using SnapGene 6.1 (https://www.snapgene.com). Additionally, the 3D structure of GBX2 was modeled using AlphaFold^11^. A phylogenetic tree illustrating the evolutionary relationships of *GBX2* among different species was generated using NCBI BLAST^®12^.

**Cell culture**

The HEK-293T cell line was obtained from Merck (Shanghai, China) and cultured in high-glucose Dulbecco's Modified Eagle Medium (Biological Industries, Beit HaEmek, Israel) supplemented with 10% fetal bovine serum (Gibco, CA, USA), and 1% Penicillin-Streptomycin Solution (NCM Biotech, Jiangsu, China) at 37℃ with 5% CO2.

**Vector constructs**

The full coding sequence of *GBX2* (NM_001485) was cloned into the pCDH vector (GeneRay, Shanghai, China) and the pXT7 vector (GeneRay, Shanghai, China), for overexpression of *GBX2* in HEK-293T cells and zebrafish, respectively. Mutant *GBX2* vectors were generated using the Q5 Site-Directed Mutagenesis Kit (E0554, New England BioLabs, Beverly, MA, USA) and subsequently verified by Sanger sequencing. The 242 kb core promoter sequence (see Supplementary Note) of *EEF1A1* was cloned into the pGL3-Basic Luciferase Reporter Vector (GeneRay, Shanghai, China) for luciferase assays.

**RNA extraction, reverse transcription and quantitative polymerase chain reaction**

Total RNA was extracted from HEK-293T cells and zebrafish using TRIzol reagent (Invitrogen, CA, USA) according to the manufacturer's protocol. RNA quality and concentration were assessed before reverse transcription to cDNA using the Prime Script RT Reagent Kit (Yeasen, Shanghai, China). Quantitative real-time polymerase chain reaction (qPCR) was performed with SYBR Premix Ex Taq™ (Takara, Shiga, Japan) on a StepOnePlusTM Real-Time PCR System (Thermo Fisher Scientific, MA, USA). The qPCR cycling conditions included an initial denaturation at 95°C for 30 seconds, followed by 40 cycles of denaturation at 95°C for 5 seconds, annealing at 60°C for 30 seconds, and extension at 95°C for 15 seconds. Relative mRNA expression was normalized to the human actin beta (ACTB) and zebrafish glyceraldehyde 3-phosphate dehydrogenase (gapdh) genes and quantified using the relative quantification method (2^−ΔΔCt^). Primers sequences are listed in **Table S3**.

**Western blot assay**

HEK-293T cells transfected with pCDH vectors were harvested and lysed using radioimmunoprecipitation assay buffer (Yeasen, Shanghai, China), supplemented with 1% phenylmethanesulfonyl fluoride (Beyotime, Shanghai, China). Protein concentrations were determined using a Bradford Protein Assay Kit (Abcam, MA, USA). Equal amounts of protein were separated by 10% SDS-polyacrylamide gel electrophoresis (NCM Biotech, Suzhou, China) , and the proteins were transferred to nitrocellulose membranes (Pall, NY, USA). The membranes were blocked with 8% skimmed milk at room temperature for 1 hour, followed by overnight incubation at 4℃ with primary antibodies: anti-FLAG (Abmart, Shanghai, China) at a 1:1000 dilution and anti-beta-tubulin (Proteintech, IL, USA) at a 1:1000 dilution.

**Luciferase assay**

To perform luciferase assays, HEK-293T cells were seeded in 96-well plates and cultured to 80% confluence. The cells were transfected with 0.25 μg of *GBX2* pCDH vectors, 0.25 μg of pGL3-Basic vector containing EF-1α core promoter, and 0.01 μg of pGL4.70 vectors using Lipofectamine 3000 transfection reagent (L3000008, Thermo Fisher, Paisley, Scotland, UK). Twenty-four hours post-transfection, cells were harvested and lysed, and the fluorescence intensity was measured using the Dual Luciferase Reporter Gene Assay Kit (11402ES10, Yeasen, Shanghai, China) following the manufacturer’s protocol.

**Analysis of spatial enhanced resolution omics sequencing data and single-cell RNA sequencing data**

We analyzed the Zebrafish Embryogenesis Spatiotemporal Transcriptomic Atlas (https://db.cngb.org/stomics/zesta/)^13^ using spatial enhanced resolution omics sequencing (Stereo-seq) data. Specifically, we selected the file “spatial six time slice stereoseg.h5ada” and input the gene name to visualize the expression pattern of *gbx2* during 3.3–24 hours post-fertilization (hpf) window. Subsequently, we analyzed the single-cell zebrafish atlas data from the zebrafish atlas^14^(GSE202639), which includes samples from 18 to 96 hpf. The raw data were processed using Monocle3 (v.1.3.1). To examine the expression pattern of *gbx2* during zebrafish craniofacial development., we divided the zebrafish global reference dataset into four major groups, as previously described^14^. We then focused on a subset of the data corresponding to pharyngeal arch cells, which are most relevant to craniofacial development. After re-processing, we used Uniform Manifold Approximation and Projection (UMAP) to embed the data in 3D space and identified distinct cell clusters by subclustering. These clusters were annotated based on the expression of marker genes, identified using the top_markers function, and cross-referenced with literature and anatomical terms from the ZFIN database (zfin.org). Finally, UMAP and dot plot visualizations were used to represent the expression of *gbx2* across these 12 cell clusters.

**Zebrafish knockdown and rescue experiments**

The *Tg(sox10:* GFP*)* transgenic AB zebrafish strain, which enables visualization of CNCCs and neural crest-derived pharyngeal cartilage, was obtained from YSY Biotech Company Ltd (Nanjing, China). Zebrafish embryos were collected from natural spawning from adult zebrafish.

For loss-of-function evaluation of *gbx2*, a modified CRISPR interference (CRISPRi) protocol was employed as previously described^15^. Briefly, single guide RNAs (sgRNAs) targeting *gbx2* were designed using CRISPRscan (http://www.crisprscan.org). Four sgRNAs were synthesized by Invitrogen (MA, USA) according to the manufacturer’s guidelines (**Table S3**). Each sgRNA was injected separately into zebrafish embryo to verify its efficiency in inducing insertions or deletions. To induce CRISPRi in zebrafish, a mixture of the four sgRNAs (400 ng/μL) and 1nL of dCas9-krab-mRNA was injected into single-cell stage embryos. In the control group, only 1nL of dCas9-krab-mRNA was injected. After injection, the embryos were cultured at 28℃ in E3 medium (containing 5 mM NaCl, 0.17 mM KCl, 0.33 mM MgSO4, 0.33 mM CaCl2, and 0.01% methylene blue) supplemented with 0.003% 1-phenyl 2-thiourea to prevent pigmentation. The qPCR was performed to confirm the successful knockdown of *gbx2*, using primers listed in **Table S3**. Zebrafish gross morphology was photographed using a dissection microscope. Body length and the ratio of head thickness to body length was measured as described by Ando et al. (2019)^16^. Specifically, body length was defined as the straight-line distance from the mouth to the tip of the tail.

To rescue *gbx2* expression in *gbx2* knockdown zebrafish, the four sgRNAs were co-injected with either a wild-type (WT) human *GBX2* expression vector (pXT7-GBX2, 150 pg/nL) or a variant expression vector (pXT7-GBX2 p.A4V or pXT7-GBX2 p.Q67H, 150 pg/nL) into one-cell stage *sox10:* GFP zebrafish embryos (1 nL per embryo).

**Bromodeoxyuridine assay**

To assess zebrafish proliferation, Bromodeoxyuridine (BrdU) assay were performed on *gbx2* knockdown and control group at 24 hpf. Briefly, embryos were incubated in E3 medium supplemented with 10 mM BrdU for 6 hours. After incubation, the embryos were fixed in 4% paraformaldehyde for 4 hours. Subsequently, the embryos were treated with 0.2 N hydrochloric acid (Sinopharm Chemical Reagent, Shanghai, China) for 1 hour at room temperature. Immunostaining was performed by incubating the fixed embryos overnight at 4℃ with anti-BrdU antibody (1:100, ABT2021, Santa Cruz Biotechnology, CA, USA) and anti-GFP Tag Rabbit Polyclonal Antibody (1:250, abbkine, Georgia, USA). The embryos were washed with phosphate-buffered saline (PBS) and then incubated with Goat Anti-Mouse IgG 488 secondary antibody (Yisheng, Shanghai, China) at a dilution of 1:200 overnight at 4℃. After washing again with PBS, the embryos were stained with DAPI (1:1000, 40728ES03, Yeasen, Shanghai, China) for 10 minutes.

**Confocal microscopy analysis**

Zebrafish embryos were mounted in 80% glycerol (Sinopharm Chemical Reagent, Shanghai, China) in 35 mm glass bottom dishes (Cellvis, CA, USA). Confocal images were acquired using a TCS SP8 confocal inverted microscope (Leica, Germany). The images were processed and analyzed using ImageJ software (v.1.52) for quantification

**Statistical analysis**

All experimental samples were prepared in triplicate, and data were normalized to their corresponding control groups. Each experiment was repeated independently three times. Statistical analysis was performed using GraphPad Software Inc. (La Jolla, CA, USA). A paired two-tailed Student’s t-test was used to compare groups, with a significance threshold of *P* < 0.05.

**Table S1. The detailed clinical information of patients carrying *GBX2* variants in the three pedigrees with CFM**

| Pedigree | | 1 | | | 2 | | 3 | |
| --- | --- | --- | --- | --- | --- | --- | --- | --- |
| Patients | | II:3* | I:1 | II:1 | II:1* | I:2 | II-1 * | I:2 |
| Age | | 4 | 40 | NA | 10 | NA | 5 | NA |
| Sex | | Male | Male | Female | Male | Female | Male | Female |
| DNA Alteration | | c.11C>T | c.11C>T | c.11C>T | c.11C>T | c.11C>T | c.201G>C | c.201G>C |
| Amino Acid Alteration | | p.A4V | p.A4V | p.A4V | p.A4V | p.A4V | p.Q67H | p.Q67H |
| Variant Zygosity | | Het | Het | Het | Het | Het | Het | Het |
| Side | | Bilateral | Bilateral | Left | Right | Right | Right | Right |
| Auricle malformation classification^17^ | R | Grade III | Grade I | Normal | Grade III | Grade I | Grade I | Grade III |
|  | L | Grade III | Grade I | Grade I | Normal | Normal | Normal | Normal |
| External auditory canal | R | Stenosis | Atresia | Normal | Stenosis | Atresia | Atresia | Stenosis |
|  | L | Stenosis | Atresia | Atresia | Normal | Normal | Normal | Normal |
| Middle ear | R | Normal | NA | NA | Affected | NA | Affected | NA |
|  | L | Normal | NA | NA | Normal | NA | Normal | NA |
| OMENS  classification^18^ | | O_0_M_1_E_3_N_0_S_3_ | / | / | O_0_M_1_E_3_N_0_S_1_ | / | O_0_M_0_E_2_N_0_S_1_ | / |

*Proband; CFM, craniofacial microsomia; Het, Heterozygous; R, Right; L, Left; NA, not available. OMENS: orbit, mandible, ear, nerve, and soft tissue

Table S1. Footnote:

Pedigree 1: The proband (II:3), a 4-year-old male, presented with bilateral Grade III microtia (Figure 1A), facial asymmetry, and severe mixed hearing loss (Figure 1B). three-dimensional computed tomography (3D-CT) imaging revealed stenosis of the external auditory canal bilaterally, while middle ear structures appeared normal (Figure 1B, S1B-C). Mandibular asymmetry was noted, with an asymmetry index (AI) of 7.8%, exceeding the clinical threshold of 5%, contributed to an orbit, mandible, ear, nerve, and soft tissue (OMENS) classification of O_0_M_1_E_3_N_0_S_3_ for the proband (Figure 1B). Other affected family members, I:1 and II:1, exhibited similar but milder phenotypes, I:1 presented with bilateral Grade I microtia and atresia of the external auditory canal, whereas II:1 exhibited left Grade I microtia and atresia of the left ear canal (Figure 1A).

Pedigree 2: The proband (II:1), a 10-year-old male, presented with right-sided Grade III microtia, facial asymmetry (Figure 1A), and severe mixed hearing loss on the right side (Figure S1A). CT imaging showed stenosis of the right external auditory canal with middle ear involvement (Figure S1B-C). Mandibular involvement was observed with an AI of 9.2%, contributed to an OMENS classification of O_0_M_1_E_3_N_0_S_1_ (Figure 1B). His mother (I:2) displayed right Grade I microtia and atresia of the right external. (Figure 1A)

Pedigree 3: The proband (II:1), a 5-year-old male, exhibited right-sided Grade I microtia, facial asymmetry (Figure 1A), and mild to moderate conductive hearing loss (Figure S1A). CT revealed atresia of the right external auditory canal and middle ear involvement with no mandible asymmetry, resulting in an OMENS classifcation of O_0_M_0_E_2_N_0_S_1_(Figure 1C, 1D, 1E). His mother (I:2) also presented with right-sided Grade III microtia and stenosis of the right auditory canal (Figure 1A).

**Table S2. The general information about the two variants in *GBX2***

| Pedigree | Variant | ExAC_ALL | GnomAD_EAS | GnomAD_ALL | Mutation Taster | Polyphen2_HDIV | CADD | Mutation Type |
| --- | --- | --- | --- | --- | --- | --- | --- | --- |
| 1,2 | c.11C>T | 1.78E-6 | 6.86E-4 | 1.67E-5 | D(0.99) | D(0.969) | 33 | Point mutation (missense) |
| 3 | c. 201G>C | 2.05E-6 | 2.54E-6 | 1.86E-6 | D(0.98) | P(0.472) | 22 | Point mutation (missense) |

*D: damaging, P: possibly damaging, CADD > 20: potential pathogenicity.

**Table S3. OMENS Classification System**

| Classification | Grade | Description |
| --- | --- | --- |
| Orbit | O_0_ | Normal orbital size and position |
|  | O_1_ | Abnormal orbital size |
|  | O_2_ | Abnormal orbital position (arrow up or down) |
|  | O_3_ | Abnormal orbital size and position |
| Mandible | M_0_ | Normal mandible |
|  | M_1_ | Smaller than preserved normal side |
|  | M_2_ | Condyle, ramus, and sigmoid notch identifiable, but grossly distorted in size and shape |
|  | M_3_ | Grossly distorted ramus with loss of landmarks or agenesis |
| Ear | E_0_ | Normal ear |
|  | E_1_ | Milhjd hypoplasia & cupping, all structures present |
|  | E_2_ | Absence of external auditory canal with hypoplasia of concha |
|  | E_3_ | Malpositioned lobule with absent auricle, lobular remnant inferiorly and anteriorly displaced |
| Facial Nerve | N_0_ | No facial nerve involvement |
|  | N_1_ | Upper facial nerve involvement (temporal zygomatic) |
|  | N_2_ | Lower facial nerve involvement (buccal, mandibular, cervical) |
|  | N_3_ | All branches of facial nerve affected |
| Soft Tissue | S_0_ | No obvious soft tissue or muscle deficiency |
|  | S_1_ | Minimal subcutaneous/muscle deficiency |
|  | S_2_ | Moderate–between the two extremes S1 and S3 |
|  | S_3_ | Severe soft tissue deficiency due to subcutaneous and muscular hypoplasia |

**Table S4. List of primer sequences**

All sequences are given in 5' to 3' direction

| Primers used for Sanger sequencing | |
| --- | --- |
| GBX2-seq-hu-1-F | GACGGACGGCAGCTTACC |
| GBX2-seq-hu-1-R | CTGGGGATCTGGTGGTGAG |
| GBX2-seq-hu-2-F | GATAAGTACGCCGGCTTCGC |
| GBX2-seq-hu-2-R | CGTAGAGGTGAGCGCCAT |
| sgRNA target sequence for generating F_0_ knockdown crispant | |
| gbx2-sgRNA1-F | TAATACGACTCACTATAGCTGGACGACGGCGTTCACGGgttttagagctagaaatagc |
| gbx2-sgRNA2-F | TAATACGACTCACTATAGCGATTTGCGAGCGTTCTGTGgttttagagctagaaatagc |
| gbx2-sgRNA3-F | TAATACGACTCACTATAGGCACGCCTTAAAGCTCAGCGgttttagagctagaaatagc |
| gbx2-sgRNA4-F | TAATACGACTCACTATAGGTTCCCGGGCCCGTTATCATgttttagagctagaaatagc |
| Primers used for qPCR | |
| GBX2-Q1-hu-F | GTACCTCTCCTTGACCGAGC |
| GBX2-Q1-hu-R | GGGACGACGATCTTAGGGTT |
| Gbx2-Q1-mo-F | CAACTTCGACAAAGCCGAG |
| Gbx2-Q1-mo-R | CCTTTGACTCGTCTTTCCCT |
| gbx2-Q1-ze-F | CCACGATTCGCAGTCCGTAC |
| gbx2-Q1-ze-R | TCTCGTCTTTCCTGTGACAA |
| gapdh-Q1-ze-F | CCAACTGCCTGGCTCCTT |
| gapdh-Q1-ze-R | CCCATCAACGGTCTTCTGTG |
| GAPDH-hu-q1-F | GGAGCGAGATCCCTCCAAAAT |
| GAPDH-hu-q1-R | GGCTGTTGTCATACTTCTCATGG |
| ACTB-hu-q1-F | CATGTACGTTGCTATCCAGGC |
| ACTB-hu-q1-R | CTCCTTAATGTCACGCACGAT |

**Supplementary Note. The 242 kb core promoter sequence of *EEF1A1***

GGTACCGCTCCGGTGCCCGTCAGTGGGCAGAGCGCACATCGCCCACAGTCCCCGAGAAGTTGGGGGGAGGGGTCGGCAATTGAACGGGTGCCTAGAGAAGGTGGCGCGGGGTAAACTGGGAAAGTGATGTCGTGTACTGGCTCCGCCTTTTTCCCGAGGGTGGGGGAGAACCGTATATAAGTGCAGTAGTCGCCGTGAACGTTCTTTTTCGCAACGGGTTTGCCGCCAGAACACAGAAGCTT

**Reference for Supplementary Methods and Materials**

1. Gougoutas AJ, Singh DJ, Low DW, Bartlett SP. Hemifacial microsomia: clinical features and pictographic representations of the OMENS classification system. *Plast Reconstr Surg.* 2007;120(7):112e-113e.

2. Hunter A, Frias JL, Gillessen-Kaesbach G, Hughes H, Jones KL, Wilson L. Elements of morphology: standard terminology for the ear. *Am J Med Genet A.* 2009;149A(1):40-60.

3. Li H, Durbin R. Fast and accurate short read alignment with Burrows-Wheeler transform. *Bioinformatics.* 2009;25(14):1754-1760.

4. McKenna A, Hanna M, Banks E, et al. The Genome Analysis Toolkit: a MapReduce framework for analyzing next-generation DNA sequencing data. *Genome Res.* 2010;20(9):1297-1303.

5. Talevich E, Shain AH, Botton T, Bastian BC. CNVkit: Genome-Wide Copy Number Detection and Visualization from Targeted DNA Sequencing. *PLoS Comput Biol.* 2016;12(4):e1004873.

6. Wang K, Li M, Hakonarson H. ANNOVAR: functional annotation of genetic variants from high-throughput sequencing data. *Nucleic Acids Res.* 2010;38(16):e164.

7. Cingolani P, Platts A, Wang le L, et al. A program for annotating and predicting the effects of single nucleotide polymorphisms, SnpEff: SNPs in the genome of Drosophila melanogaster strain w1118; iso-2; iso-3. *Fly (Austin).* 2012;6(2):80-92.

8. Schwarz JM, Cooper DN, Schuelke M, Seelow D. MutationTaster2: mutation prediction for the deep-sequencing age. *Nat Methods.* 2014;11(4):361-362.

9. Adzhubei IA, Schmidt S, Peshkin L, et al. A method and server for predicting damaging missense mutations. *Nat Methods.* 2010;7(4):248-249.

10. Kircher M, Witten DM, Jain P, O'Roak BJ, Cooper GM, Shendure J. A general framework for estimating the relative pathogenicity of human genetic variants. *Nat Genet.* 2014;46(3):310-315.

11. Jumper J, Evans R, Pritzel A, et al. Highly accurate protein structure prediction with AlphaFold. *Nature.* 2021;596(7873):583-589.

12. Altschul SF, Gish W, Miller W, Myers EW, Lipman DJ. Basic local alignment search tool. *J Mol Biol.* 1990;215(3):403-410.

13. Liu C, Li R, Li Y, et al. Spatiotemporal mapping of gene expression landscapes and developmental trajectories during zebrafish embryogenesis. *Dev Cell.* 2022;57(10):1284-1298 e1285.

14. Saunders LM, Srivatsan SR, Duran M, et al. Embryo-scale reverse genetics at single-cell resolution. *Nature.* 2023;623(7988):782-791.

15. Long L, Guo H, Yao D, et al. Regulation of transcriptionally active genes via the catalytically inactive Cas9 in C. elegans and D. rerio. *Cell Res.* 2015;25(5):638-641.

16. Ando H, Sato T, Ito T, et al. Cereblon Control of Zebrafish Brain Size by Regulation of Neural Stem Cell Proliferation. *iScience.* 2019;15:95-108.

17. Zhang TY, Bulstrode N, Chang KW, et al. International Consensus Recommendations on Microtia, Aural Atresia and Functional Ear Reconstruction. *J Int Adv Otol.* 2019;15(2):204-208.

18. Birgfeld CB, Heike C. Craniofacial microsomia. *Semin Plast Surg.* 2012;26(2):91-104.
